# mSphere of Influence: From pixels to parasite insight—cryo-EM is rewiring apicomplexan cell biology

**DOI:** 10.1128/msphere.00231-25

**Published:** 2025-08-11

**Authors:** Li-av Segev-Zarko

**Affiliations:** 1 Department of Biochemistry and Molecular Biology, The University of Texas Medical Branch 12338, Galveston, Texas, USA; Virginia-Maryland College of Veterinary Medicine, Blacksburg, Virginia, USA

**Keywords:** cryo-EM, apicomplexan parasites, resolution revolution

## Abstract

Li-av Segev Zarko studies the nanomachines that apicomplexan parasites deploy to break into host cells. In this mSphere of Influence article, she reflects on how breakthrough cryo-electron microscopy studies published between 2018 and 2021 reshaped her view of what the ever-advancing field of structural biology can reveal about molecular and cellular parasitology.

## TRACING CLARITY THROUGH A CENTURY OF ELECTRON MICROSCOPY

Electron microscopy (EM) has illuminated protozoan parasites for almost a century. Soon after Ruska and Knoll built the first transmission EM, Gustafson et al. (1) imaged *Toxoplasma gondii*, revealing the distinctive morphology of organelles we now commonly recognize (1). Since then, freeze-fracture and resin-embedded sections have been indispensable for identifying and naming intracellular structures, yet they lacked the high-resolution structural detail needed to reveal how they worked. Cryo-EM’s “resolution revolution” promised to lift that fog— and several papers published between 2018 and 2021 advanced toward that goal. Each study introduced a new tier of resolution or context, and together, they demonstrated that contemporary structural biology can trace a parasite from its isolated subunits to the active nanomachinery operating inside a living cell.

## SNAPSHOTS THAT SHIFTED THE FRAME

The first jolt came from Ho et al. (2), who solved the *Plasmodium falciparum* protein-export translocon (PTEX) at 3.2 Å (2). The authors used cryo-EM, specifically single-particle analysis, to overcome the limitations of traditional methods and resolve the global architecture of the mysterious, megadalton-sized membrane machine at near-atomic resolution. By purifying PTEX endogenously, Ho et al. provided superior mechanistic insight that recombinant subunits might not capture. Next, Wang et al. (3) targeted the parasite cytoskeleton, solving the structure of *Toxoplasma* cortical microtubules at 4 Å (3). By comparing the cryo-EM globular densities to a library of protein domains, the authors identified candidate structures that were then docked back into the density map, resolving the identity of previously mysterious luminal blobs lining the microtubule interior. By building an atomic model of the entire complex, Wang et al. provided the structural basis for understanding the remarkable stability of cortical microtubules. If PTEX delivered detail and cortical microtubules revealed new identities, Aquilini et al. provided context. The authors plunge-froze intact tachyzoites and used cryo-electron tomography (cryo-ET) to resolve an eightfold symmetric rosette embedded at the apex of *Toxoplasma* and its association with the secretory organelles known as rhoptries (4). Building on this, Mageswaran et al. (5) applied cryo-ET and subtomogram averaging to show that depleting ND9—a gene required for rhoptry secretion—disrupts the rosette’s symmetry (5). Together, these studies connected cellular and molecular structure to function, presenting a model for the rhoptry secretion system.

## BRINGING MY OWN FRAME INTO FOCUS

Not long after I began my postdoc, these papers were published, showcasing cutting-edge technological advances and delivering pivotal insights into the field of cellular and molecular parasitology. Together, they offered a conceptual ladder that continues to guide my lab: (i) elusive targets are solvable (Ho), (ii) near-atomic maps can reveal entirely new proteins (Wang), and (iii) high-resolution structural context can be resolved *in situ* under near-native conditions (Aquilini and Mageswaran). Conceptually, these studies helped dissolve the wall between structural and cell biology. In practice, we now combine these approaches with focused-ion-beam milling to overcome sample thickness limitations and explore how the structure and context of nanometer-scale machines shape their function.

## TOWARD A MOVING PICTURE

While the leap from traditional TEM to cryo-ET was dramatic, the next frontier lies in temporal resolution. Time-resolved plunge freezing, combined with on-grid stimulation, promises to transform single snapshots into molecular movies. Coupled with AI-driven image analysis pipelines to push *in situ* resolution below 4 Å, this approach will unite atomic detail, cellular context, and real-time dynamics in a single workflow. When that happens, cellular processes will evolve from a single still frame to a full-length feature film—one high-definition scene at a time.
